# Assessment of pathogen removal efficiency of vertical flow constructed wetland treating septage

**DOI:** 10.1038/s41598-023-45257-2

**Published:** 2023-10-31

**Authors:** Swati Singh, Sweta Upadhyay, Anju Rani, Pradeep Kumar Sharma, Janhvi Mishra Rawat, Balwant Rawat, Prashant, Prosun Bhattacharya

**Affiliations:** 1 Department of Environmental Science, Graphic Era (Deemed to be University), Dehradun, Uttarakhand India; 2 Department of Microbiology, Graphic Era (Deemed to be University), Dehradun, Uttarakhand India; 3 Department of Biotechnology, Graphic Era (Deemed to be University), Dehradun, Uttarakhand India; 4 School of Agriculture, Graphic Era Hill University, Dehradun, Uttarakhand India; 5 Department Environmental Science, Central University of South Bihar, Gaya, Bihar India; 6 Department of Sustainable Development, Environmental Sciences and Engineering, KTH Royal Institute of Technology, Teknikringen 10B, 100 44 Stockholm, Sweden; 7 KWR Water Cycle Research Institute, Groningenhaven 7, 3433 PE Nieuwegein, the Netherlands

**Keywords:** Applied microbiology, Biofilms, Microbial communities, Environmental microbiology, Pathogens, Microbiology, Environmental sciences

## Abstract

Septage refers to the semi-liquid waste material that accumulates in septic tanks and other onsite sanitation systems. It is composed of a complex mixture of human excreta, wastewater, and various solid particles. Septage is a potential source of water pollution owing to presence of high organic content, significant pathogen concentrations, and a range of nutrients like nitrogen and phosphorus. The harmful impacts of septage pollution poses significant risks to public health through the contamination of drinking water sources, eutrophication of water bodies and spread of water borne diseases. Conventional septage treatment technologies often face limitations such as high operational costs, energy requirements, and the need for extensive infrastructure. Therefore, with an aim to treat septage through an alternative cost effective and energy-efficient technology, a laboratory-scale constructed wetland (CW) system (0.99 m^2^) consisting of a sludge drying bed and a vertical flow wetland bed was utilized for the treatment of septage. The sludge drying bed and vertical flow beds were connected in series and filled with a combination of gravel with varying sizes (ranging from 5 to 40 mm) and washed sand. *Canna indica* plants were cultivated on both beds to facilitate phytoremediation process. The system was operated with intermittent dosing of 30 Ltrs of septage every day for 2 months. The HRT of the system was fixed at 48 h. The average inlet loads of Biochemical Oxygen Demand (BOD_5_), Chemical Oxygen Demand (COD), and Total Suspended Solids (TSS) were measured as 150 ± 65.7 g m^−2^ day^−1^, 713 ± 443.9 g m^−2^ day^−1^, and 309 ± 66.3 g m^−2^ day^−1^, respectively. After treatment, the final effluent had an average load of 6 g m^−2^ day^−1^ for BOD_5_, 15 g m^−2^ day^−1^ for COD, and 51 g m^−2^ day^−1^ for TSS, indicating that the CW system achieved an average removal efficiency of 88% for BOD, 87% for COD, and 65% for TSS. The average load of total coliforms and helminthes eggs in the influent was recorded as 4 × 10^8^ Colony-Forming Units (CFU) m^−2^ day^−1^ and 3 × 10^7^ eggs m^−2^ day^−1^, respectively. However, the CW system demonstrated significant effectiveness in reducing microbial contamination, with an average removal efficiency of 99% for both total coliforms and helminthes eggs. The vertical flow constructed wetland system, equipped with pretreatment by sludge drying bed, has proven to be efficient in treatment of septage.

## Introduction

Septage, also known as septage sludge or septic tank effluent, refers to the semi-liquid waste material that accumulates in septic tanks and other on-site sanitation systems. It contains a mixture of human excreta, wastewater, and solid particles^1^. The characteristics of septage comprise high organic content, significant pathogen concentrations^2^, and the presence of nutrients like nitrogen and phosphorus. India generates a significant amount of septage owing to its vast population and the widespread use of on-site sanitation systems. However, the treatment capacity for septage in the country remains inadequate, leading to the improper disposal of a significant portion of this wastewater.

Untreated septage is released into rivers and streams in India, causing them to deteriorate and become unfit for use. Septage contains numerous pathogens such as various coliforms^1,2^ and helminthes eggs^3,4^ which have detrimental effects on human health once they reach into the body. These pathogens are difficult to remove by conventional wastewater treatment methods^5^. Various methods are employed for septage treatment in India, including traditional systems like septic tanks and pit latrines, co-treatment with sewage, as well as more advanced approaches such as decentralized treatment plants, biological sand filters, biofilters and constructed wetlands (CWs)^6^. But CWs are favorable because of their ease of operation, low cost establishment and better ability to remove microbial and organic pollutants. Furthermore, constructed wetland (CW) technology is a preferred technology for septage treatment in developing and populated countries where it is difficult to set up large scale infrastructure due to high cost and requirement of larger area for wastewater treatment plant. Constructed wetlands are well known for their ability to utilize natural processes^7^ for purification of wastewater while effectively removing contaminants. Several types of constructed wetland systems such as free water surface wetlands and sub-surface flow wetlands have been tested in the past for treatment of variety of wastewater, however, sub-surface flow CW systems are preferred over free water surface wetlands due to their reduced footprints and better efficiency. Among the sub-surface flow CW designs, most popular designs are vertical flow^8–10^, horizontal flow^11,12^, tidal flow and hybrid designs of CWs. Constructed wetlands have established their effectiveness in the removal of pathogens and various pollutants^4,5^. In the recent past, vertical flow CW systems have gained more attention for their effectiveness in septage treatment. These systems consist of beds filled with coarse media (e.g., gravel) and river sand where septage is introduced from the top and percolates vertically downward through the substrate. Key advantages of vertical flow constructed wetlands for septage treatment includes compact footprints, enhanced nutrient removal, energy efficient and aesthetically pleasing systems. Along with this, vertical sub-surface flow (VSSF) constructed wetland systems (CW_S_) are also widely accepted because of their better pathogens removal efficiency^8,9,13^ and ability to offer favourable oxygen transfer conditions for nitrification of wastewater^14^. Nonetheless vertical flow systems are associated with the risks of clogging of the substrate over time, which may require periodic maintenance, and the need for appropriate pre-treatment to remove large solids and prevent clogging.

This study sought to treat septage utilizing a sludge drying bed (SDB) and vertical flow (VSSF) CW system. The treatment efficiency of the system was assessed through its potential for elimination of pathogens and organic contaminants from the septage^14–16^. Studies focusing on septage treatment with the help SDB-VSSF design are limited however few studies in the past focused on treatment of septage by utilizing VSSF or SDB separately but none of them used both of these systems altogether for removal of helminthes eggs and total coliform along with TDS, TSS, BOD_5_ and COD^17,18^. This study helps to bridge the knowledge gap regarding removal of helminthes eggs and total coliform by implementing a CW system comprising SDB and VSSF.

## Methodology

### Layout and construction of laboratory-scale vertical subsurface flow (VSSF) CW system

**Figure 1:**
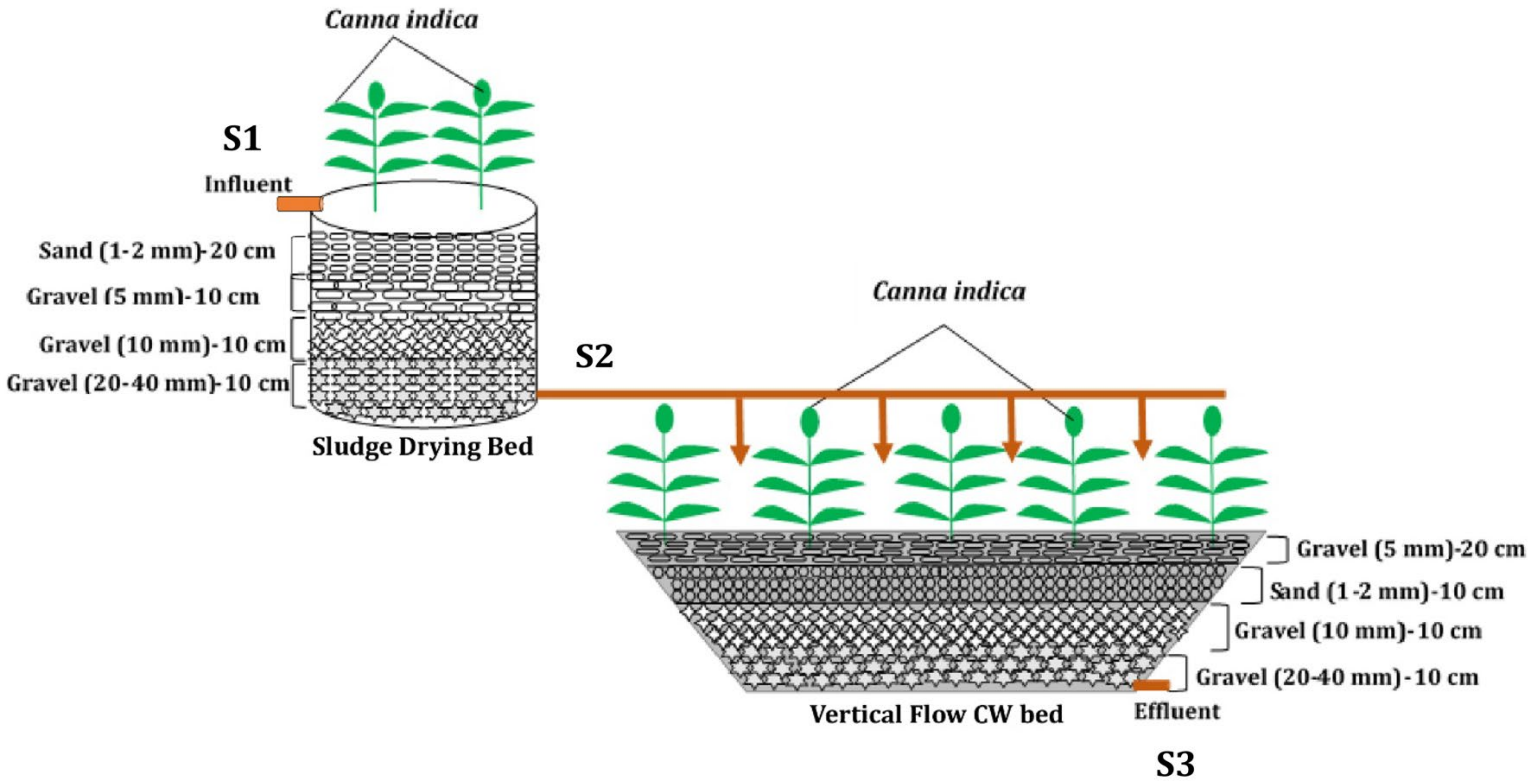
Schematic diagram of constructed wetland system for treatment of Septage.

A lab-scale vertical sub-surface flow (VSSF) constructed wetland (CW) system was implemented, at Graphic Era University's research field station, comprising a sludge drying bed^19^ and a vertical flow CW bed^20^ (Fig. 1). These beds were interconnected in series, sloping at a 1% gradient from the inlet to the discharge point. Each day, approximately 30 L of septage underwent treatment in the CW system using an intermittent dosage regimen.

Both beds were filled with sand and gravel of varying sizes. The sludge drying bed (SDB) was specifically layered, starting with a topmost 20 cm layer of sand followed by three subsequent layers, each 10 cm thick, consisting of gravel with diameter of 5 mm, 10 mm, and 20–40 mm, respectively. A cylindrical plastic container with a diameter of 41.5 cm was utilized as the SDB, which had a total area of 0.41 m^2^ and a depth of 50 cm. A PVC drainage pipe was placed at the bed's base for the efficient discharge of treated wastewater, and the hydraulic retention time (HRT) for the SDB was set at a fixed 24 h. *Canna indica* was grown over the bed surface for facilitating the phytoremediation process and maintaining aerobic conditions.

Subsequently, a vertical subsurface flow (VSSF) bed was constructed using an HDPE sheet measuring 0.76 m × 0.76 m × 0.53 m, providing a total surface area of 0.58 m^2^. Like the SDB, the VSSF bed was also operated with a HRT of 24 h and featured four distinct layers, from top to bottom. The uppermost layer (20 cm) comprised 5 mm gravel, while the following three layers, each 10 cm in thickness, consisted of 1–2 mm sand, 10 mm gravel, and 20 mm gravel, respectively. The entire constructed wetland (CW) system covered a combined area of 0.99 m^2^. Similar to SDB, *Canna indica* was grown over VSSF bed also. *Canna indica* specimens were obtained from the University's agricultural experimental station and underwent rigorous identification by a taxonomy expert at the Uttarakhand State Application Centre (USAC). Prior to transplanting them into wetland beds, the plants were thoroughly washed with tap water to eliminate any impurities. They were then nurtured in a nursery for a period of three weeks to ensure their robust acclimatization to the wetland environment. The application of septage was initiated three weeks after the *Canna indica* plants had been successfully established, allowing them ample time to adapt to their new surroundings. The *Canna indica L.* showed a profound growth and was not harvested from the wetland beds during the experimental period.

Both the sand and gravel in these beds synergistically employed physical, chemical, and biological processes to effectively eliminate various contaminants, including pathogens and pollutants. These mechanisms included filtration, sedimentation, biological degradation via biofilm formation, adsorption of metal ions, chemical precipitation.

### Sample collection and analysis

Consecutive samples were taken at the S1, S2, and S3 sampling sites. Septage was collected daily and subjected to drying on a sludge drying bed (SDB) using a collection tank. The septage remained in the bed for 24 h before being transferred to the subsequent vertical flow bed. The hydraulic retention time (HRT) for the vertical flow bed was set at 24 h. The physico-chemical tests (BOD_5_, COD, TSS, TDS) and biological tests (total coliform and helminth eggs) were conducted on the samples at the Environmental Science Laboratory at Graphic Era (Deemed to be University) in Dehradun, India (30.3165° N, 78.0322° E). Entire experiment was carried out for 60 days. TDS was measured using a calibrated EC-TDS Meter (CON100, Patsio), TSS was determined using a colorimetric method, BOD_5_ was assessed using a 5-day incubation method, COD was analyzed using a reactor digestion method, total coliforms were measured by MPN method as described by US EPA^21^, and helminthes eggs were detected using a modified Bailenger method as discussed by Mahvi et al.^22^.

To identify statistically significant differences among the various parameters, including BOD_5_, COD, TSS, TS, TC, and HE, statistical analyses were performed on the acquired results. Pearson correlation matrix analysis was performed on the acquired results using OrginPro 8.5 SR1 (Origin Lab Corporation, Northampton, MA, USA) and Microsoft Excel-2019.

### Pollutant removal efficiency of CW system

The pollutant removal effectiveness of the hybrid-CW was assessed by calculating the purification and removal rates for multiple selected parameters over a six-month period from January to December 2021. The purification rate (%) was determined using the formula:

1 $${\text{Purification rate }}\left( \% \right) \, = \, \left\{ {\left( {{\text{Ci }} - {\text{ Co}}} \right)/{\text{Ci}}} \right\} \, *{ 1}00$$

where Ci and Co represent the inlet and outlet concentrations of the parameter in milligrams per liter (mg L^−1^).

Similarly, the removal rate (%) was calculated using the formula:

2 $${\text{Removal rate }}\left( \% \right) = \left\{ {\left( {{\text{Li }} - {\text{ Lo}}} \right)/{\text{Li}}} \right\} \, *{ 1}00$$

where Li represents the inlet load in grams per square meter per day (g m^−2^ day^−1^), and Lo represents the outlet load in grams per square meter per day (g m^−2^ day^−1^).

The load (L) was determined using the equation:

3 $${\text{Load }}\left( {\text{L}} \right) \, = {\text{ Q }}*{\text{ C}}/{\text{A}}$$

Here, Q represents the flow rate measured in cubic meters per day (m^3^ day^−1^), C denotes the concentration of the parameter in mg L^−1^, and A represents the area in square meters (m^2^).

Total coliform and Helminthes eggs loads were measured individually for SBD and VSSF by following formulae:

4 $${\text{Total coliforms }}\left( {{\text{CFU m}}^{{ - {2}}} {\text{d}}^{{ - {1}}} } \right) \, = \, \left\{ {\left( {{\text{Inlet CFU L}}^{{ - {1}}} - {\text{ Outlet CFU L}}^{{ - {1}}} } \right)*{\text{Dilution factor}}} \right\}/{\text{Area }}\left( {{\text{m}}^{{2}} } \right){\text{ day}}$$

5 $${\text{Helminthes eggs }}\left( {{\text{eggs m}}^{{ - {2}}} {\text{d}}^{{ - {1}}} } \right) \, = \, \left\{ {\left( {{\text{Inlet eggs L}}^{{ - {1}}} - {\text{ Outlet eggs L}}^{{ - {1}}} } \right)*{\text{Dilution factor}}} \right\}/{\text{Area }}\left( {{\text{m}}^{{2}} } \right){\text{ day}}$$

### Statement of experimental research and field studies on plant

Experimental research and field studies involving plants, including the collection of plant material, strictly adhere to the relevant guidelines and legislation at the institutional, national, and international levels. Permission was obtained from the University administration to collect and utilize *Canna indica* from the University's agricultural experimental station for experimental work. The identification of the *Canna indica* plant sample was performed by Dr. Gajendra Singh, a Scientist at USAC, India.

## Results and discussion

### BOD_5_ and COD load and removal rate

**Figure 2:**
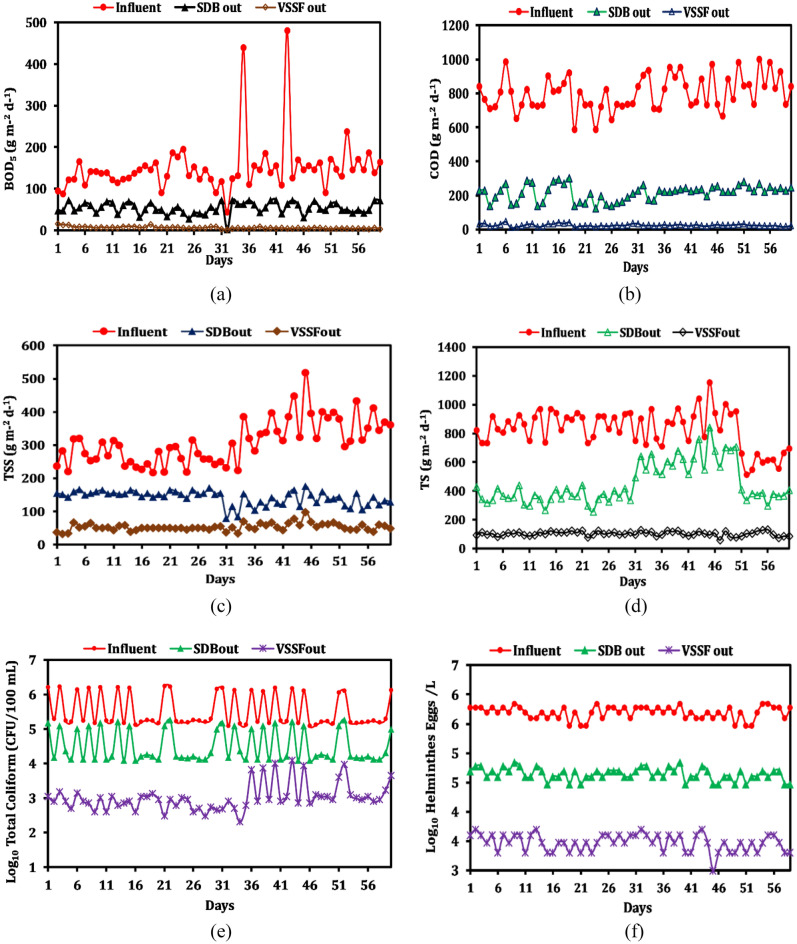
Dynamics of (**a**) BOD, (**b**) COD, (**c**) TSS, (**d**) TS, (**e**) total coliforms, and (**f**) total helmminthes in influent, and effluents of SDB, and VSSF, with respect to HRT (days).

**Figure 3:**
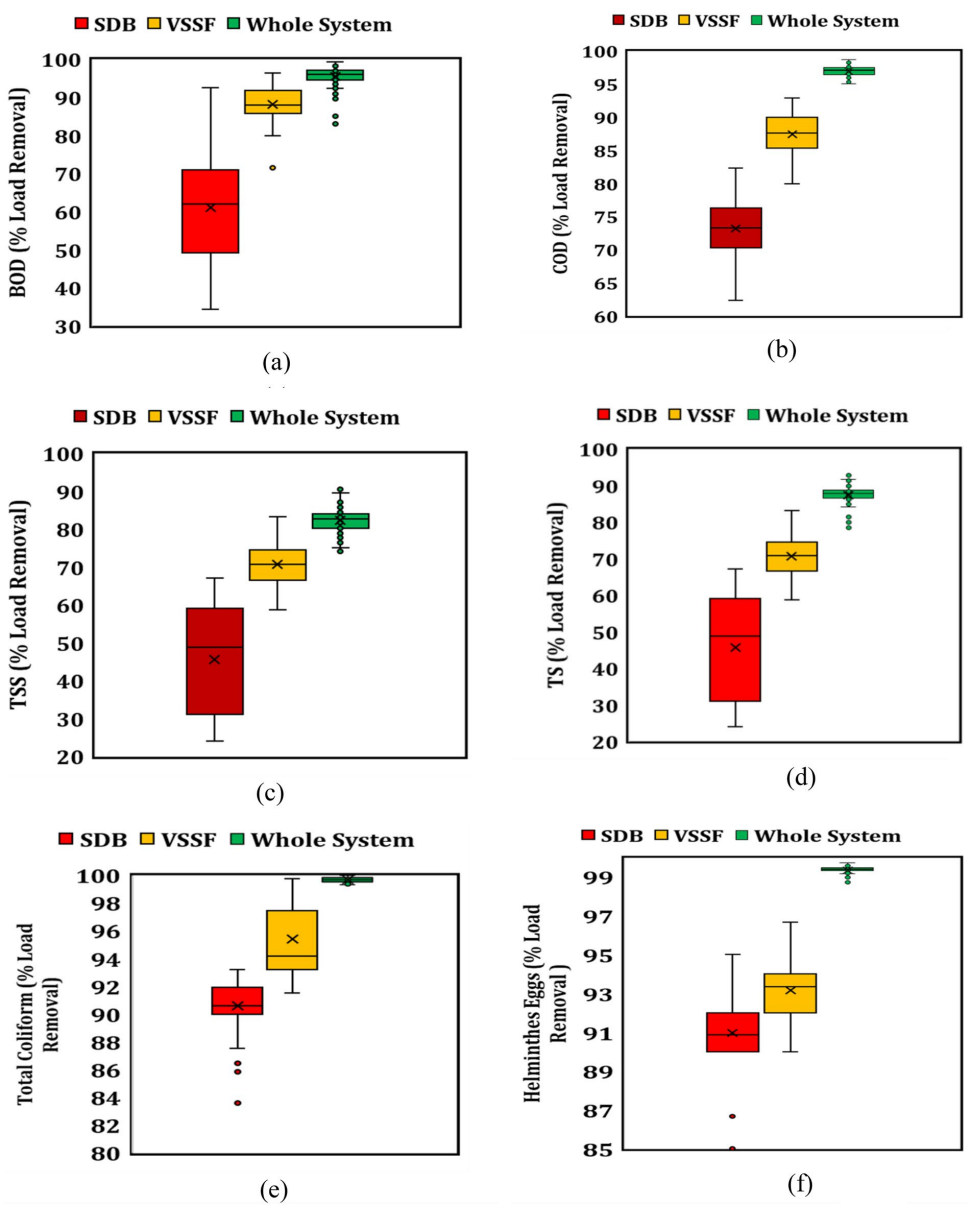
Performace of SDB, VSSF, and whole system in percentage load removal of (**a**) BOD, (**b**) COD, (**c**) TSS, (**d**) TS, (**e**) total coliforms, and (**f**) total helmminthes.

The combination of a vertical subsurface flow (VSSF) bed and a sludge drying bed (SDB) has shown remarkable improvement in the removal efficiency of the BOD_5_ load. The entire system achieved an average reduction of 93.4 ± 4.1% for BOD_5_. Despite fluctuations in the influent BOD_5_ load, ranging from 43.9 to 480.5 g m^−2^ day^−1^, both the SDB and VSSF beds effectively contributed to the removal of organic pollutants (Fig. 2a). The SDB exhibited an average BOD_5_ removal efficiency of 61.1 ± 13.5% with an average effluent load of 54.1 ± 13.96 g m^−2^ day^−1^, while the VSSF bed demonstrated a higher average BOD_5_ removal efficiency of 88.1 ± 4.9% with an average effluent load of 6.1 ± 2.72 g m^−2^ day^−1^ (Fig. 3a).This signifies a noteworthy reduction in comparison to earlier studies, ranging from 67.0% achieved by Calderón-Vallejo et al.^23^ in 2015 to 88.0% reported by Gholipour and Stefanakis^24^, demonstrating a substantial improvement in the outcomes.

The significant increase (approximately 27%) in the BOD_5_ removal efficiency of the VSSF bed compared to the SDB can be attributed to two factors: the variation in inlet BOD_5_ dosing loads and the presence of more aerobic conditions in the VSSF bed. The varying inlet BOD_5_ dosing loads likely contributed to the higher removal efficiency observed in the VSSF bed. Additionally, the design of the VSSF bed promotes a favorable aerobic environment, facilitating greater degradation of organic matter and further enhancing its performance in BOD_5_ removal.

The treatment procedure consistently resulted in a decrease in the initial BOD_5_ load, as indicated by the average BOD_5_ load of the effluent (3.6 ± 1.59 g m^−2^ day^−1^) compared to the influent load (62.1 ± 27.19 g m^−2^ day^−1^) for the entire system. This signifies a significant reduction in the amount of organic matter present. The microbial degradation of organic compounds in the wastewater as it passed through the beds contributed to this reduction. The efficient functioning of the treatment system in reducing the organic load of the influent wastewater was demonstrated by the BOD_5_ removal.

In addition to BOD_5_, the measurement of Chemical Oxygen Demand (COD) was conducted. Chemical reactions and adsorption processes played a crucial role in reducing the non-biodegradable portion of COD. The influent COD load exhibited less variability compared to BOD_5_, ranging from 241.9 to 413.4 g m^−2^ day^−1^ (Fig. 2b). The average COD load in the influent was 331.9 ± 41.5 g m^−2^ day^−1^, approximately five times higher than the average BOD_5_ load. The system achieved a significant COD load reduction with a removal efficiency of 95.7 ± 1.1%, while the SDB and VSSF beds individually achieved removal efficiencies of 73.2 ± 4.7% and 87.4 ± 3.3%, respectively (Fig. 3b). The average COD load in the SDB effluent was 215.4 ± 45.5 g m^−2^ day^−1^, while in the VSSF effluent it was 24.4 ± 6.9 g m^−2^ day^−1^. Notably, there were no significant differences observed in the COD and BOD_5_ removal efficiencies of the VSSF bed. Our research findings align well with those of Koottatep et al^20^, where they employed three pilot-scale vertical flow CW beds. These beds were filled with a sand-gravel substrata and were planted with narrow-leaf cattails (*Typhaaugustifolia*) for the treatment of septage. In their study, the CW systems were subjected to TS loading ranging from 219 to 1370 g m^−2^ day^−1^, and they observed removal rates of 80% for TS and 90% for COD. Our findings outperform the another study conducted by Calderón-Vallejo et al.^23^, which employed a similar design for septage treatment and achieved a 71% COD removal rate.

By integrating SDB and VSSF beds, the strengths of each bed can be capitalized on to achieve synergistic effects. The SDB system can serve as an effective primary treatment stage, significantly reducing the initial COD load for the subsequent VSSF system.

### TSS and TS load and removal rate

The treatment process demonstrated effective removal of suspended solids, indicating the system's efficiency in physically filtering and settling solid particles. The system achieved removal efficiencies of 74.8 ± 4.5% for TSS and 82.0 ± 3.9% for TS. The dynamics of TSS and TS removal are illustrated in Fig. 3c,d, respectively. Our system demonstrated a significantly higher efficiency in removing total solids (TS) compared to the study by Calderón-Vallejo et al.^23^, who investigated the performance of sludge drying reed beds (SDB) at both full- and pilot-scale for treating septic tank sludge in Brazil. In their study, the treatment units consisted of a sludge drying bed followed by a French vertical-flow constructed wetland. Notably, our system operated with a TS loading rate that was 2.6 times higher than the TS load in Calderón-Vallejo et al.'s study (49.3 g m^−2^ day^−1^). Despite this higher loading rate, our system achieved an impressive TS removal rate of approximately 82%, surpassing the 44% total solids removal rate reported in Calderón-Vallejo et al.'s study, which utilized a similar design of a constructed wetland system.

The influent TSS load ranged from 90.5 to 214.7 g m^−2^ day^−1^ (Fig. 2c), while the TS load ranged from 212.3 to 477.1 g m^−2^ day^−1^ (Fig. 2d). The average TSS and TS loads in the influent were recorded as 128.2 ± 27.4 g m^−2^ day^−1^ and 342.7 ± 54.7 g m^−2^ day^−1^, respectively (Table 1). Interestingly, the average TS load in the influent was approximately three to four times higher than the TSS load, indicating a significant portion of pollutants in a dissolved state. The SDB played a crucial role in removing larger particulate matter and attained an average removal rate of 46 ± 14% for TSS and 67 ± 14.1% for TS, while the VSSF bed facilitated the settling and retention of finer suspended solids. In our study, the sludge drying bed yielded a lower TSS removal rate in comparison to the research conducted by Vincet et al.^25^, in which an impressive TSS removal rate of 87.5% was achieved while treating septage using a sludge drying bed with an inlet TSS loading ranging from 82 to 137 g m^−2^ day^−1^. In a separate investigation conducted by Troesch et al.^19^, sludge drying beds that were planted with *Phragmites australis* were employed for treatment of septage at a TSS loading rate of 82 g m^−2^ day^−1^ demonstrated a remarkable TSS removal rate of 96%. In an another comparable study conducted by Kim et al.^26^, a similar design of a CW system, consisting of sludge drying bed and vertical flow bed connected in series, was examined for the treatment of septage. In this setup, the sludge drying bed was operated with an inlet TSS loading ranging from 66 to 101 g m^−2^ day^−1^, and it exhibited an exceptional TSS removal rate of 99.5%.

**Table 1:** Average ± SD load of pollutants, total coliforms and total helminthes eggs in influent, and effluents of SDB and VSSF (each data is average of 60 day’s observation), n = 3 (each day triplicate measurement was done).

Parameter	Influent	SDB out	VSSF out
BOD_5_ (g m^−2^ day^−1^)	150 ± 65.7	54.1 ± 14.0	6.1 ± 2.7
COD (g m^−2^ day^−1^)	713.4 ± 443.9	16.4 ± 9.65	15.0 ± 12.6
TSS (g m^−2^ day^−1^)	309.9 ± 66.3	143.2 ± 20.8	50.9 ± 17.1
TS (g m^−2^ day^−1^)	830.4 ± 130.3	413.5 ± 177.8	87.5 ± 37.7
Total coliforms* (CFU m^−2^ day^−1^)	4.3 × 10^8^ ± 4.5 × 10^8^	3.9 × 10^7^ ± 4.2 × 10^7^	9.0 × 10^5^ ± 1.3 × 10^6^
Helminthes* (eggs m^−2^ day^−1^)	3.7 × 10^7^ ± 8.3 × 10^6^	3.3 × 10^6^ ± 7.6 × 10^5^	1.6 × 10^5^ ± 5.0 × 10^4^

The VSSF bed demonstrated slightly superior performance in TSS removal (70.7 ± 5.8%) compared to TS removal (65.3 ± 7.1%). The average TS load in the effluents of the SDB and VSSF beds was observed as 143.2 ± 20.8 g m^−2^ day^−1^ and 54.1 ± 11.1 g m^−2^ day^−1^, respectively (Table 1).

Under the experimental conditions, the VSSF bed alone was capable of removing TSS and TS with average removal efficiencies of 70.7 ± 5.8% and 65.3 ± 7.1%, respectively, which were significantly higher than those achieved by the SDB (51.8 ± 12.1% for TSS and 45.6 ± 14.2% for TS), as shown in Fig. 3c,d. These results align with previous studies^27,28^ that highlight the role of the VSSF bed in TS and TSS removal. Tan et al.^27^ utlized a vertical flow constructed wetland (CW) system for septage treatment and demonstrated a notable TS removal rate exceeding 90%. The bed filter substrate utilized in their study comprised of coarse gravel with sizes of 50–60 mm at the bottom (20 cm), 30–45 mm gravel in the middle (30 cm), and 8–10 mm gravel at the top (30 cm), resulting in a total bed depth of 80 cm. In contrast, our vertical flow system operated at a shallower depth of 50 cm and employed finer gravel sizes compared to Tan et al.'s system. Despite these differences, we achieved a respectable TS removal rate of 82%. Our system utilized gravel sizes ranging from 5 mm in diameter to medium and coarse gravel sizes between 10 and 40 mm in diameter. Additionally, we incorporated an additional layer of sand to further enhance the TS removal efficiency in the shallow vertical flow bed. The findings indicate that the combination of the SDB and VSSF effectively removes solid particles, improving water clarity and reducing the potential for sedimentation in receiving water bodies.

### Total coliform and helminthes eggs load and removal rate

The assessment of a system's ability to provide microbiologically safe water is crucial, with a strong focus on the removal of total coliform bacteria and helminthes eggs. These contaminants pose substantial risks to public health and environmental quality, especially in wastewater. The entire system demonstrated exceptional performance, achieving a removal efficiency of over 99% for both coliform bacteria and helminthes eggs. The SDB alone exhibited an average removal of 91 ± 2% and 92 ± 3% (see Fig. 3e,f) of the initial influent counts of total coliforms (log_10_ 6 ± 5 CFU/100 mL) and helminthes eggs (log_10_ 6 ± 5 eggs L^−1^), as shown in Fig. 2e,f.

The Sludge Drying Bed (SDB) played a crucial role in effectively eliminating both bacteria and helminthes eggs, reducing their load from the septage. The high removal efficiency of the SDB can be attributed to the gradual filtration of septage through sand and multiple layers of differently sized gravels. The effluent from the SDB exhibited average counts of 4 for total coliforms and 5 for helminthes eggs (on a log10 scale).

The configuration of the VSSF bed, including the filter depth, significantly influenced the removal of pollutants and pathogens. It demonstrated the ability to remove 95 ± 3% and 93 ± 1% (Fig. 3e,f) of total coliforms and helminthes eggs, respectively. It is noteworthy that despite fluctuations in the counts of total coliforms and helminthes eggs, both beds performed equally well in reducing the microbial counts.

### Correlation study

**Figure 4:**
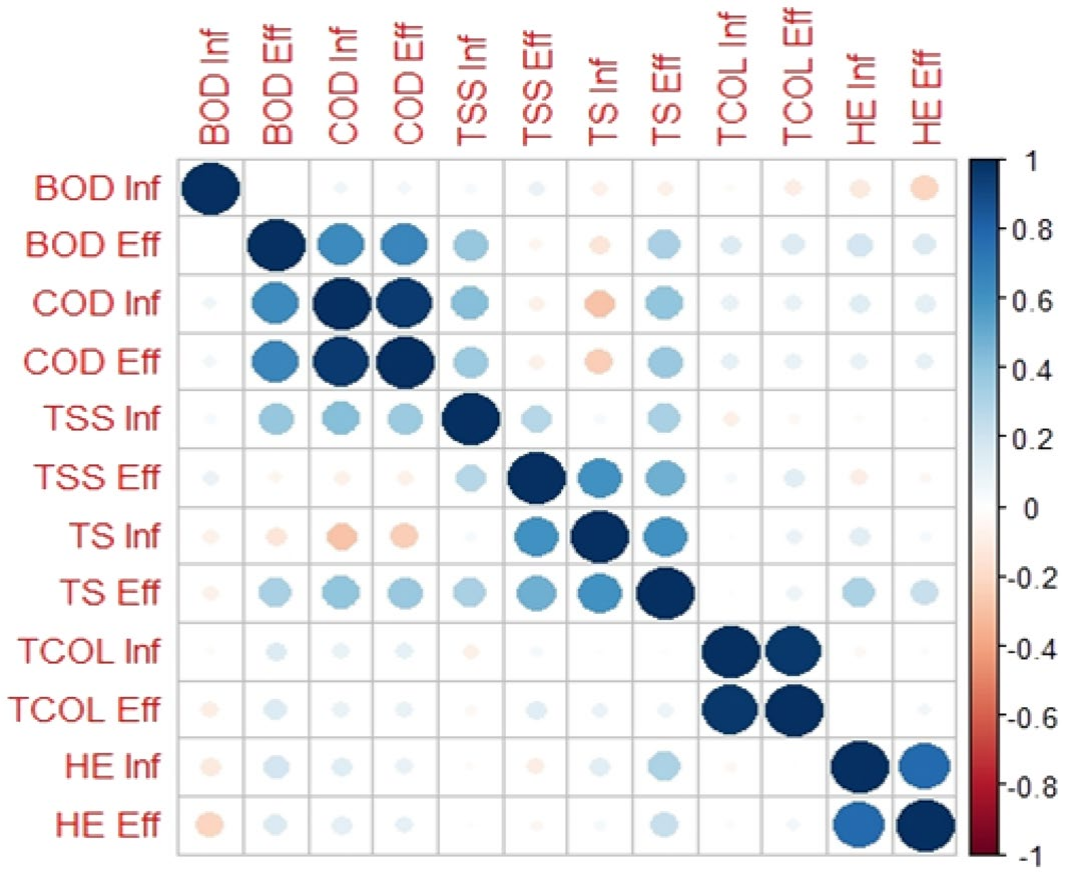
Matrix-correaltion studies (influent to effluent) of BOD_5_, COD, TSS, TS, total coliform, and total helminthes.

The correlation matrix (Fig. 4) of the entire system illustrates the interrelationships among key water quality parameters, such as COD, BOD_5_, TSS, TS, total coliform (TCOL), and helminthes eggs (HE), spanning from influent to effluent. The correlation coefficients offer valuable insights into the strength and direction of these relationships, providing a comprehensive understanding of the interconnectedness of these parameters.

In the correlation matrix encompassing the entire system, notable positive correlations were identified between the influent and effluent values for the same parameters. These correlations include COD inf vs. COD eff (r = 0.99), TCOL eff vs. TCOL inf (r = 0.996), HE eff vs. HE inf (r = 0.85), and TS inf vs. TS eff (r = 0.66). These results indicate that the composition of the influent significantly influences the quality of the effluent, emphasizing the effectiveness of the SDB and VSSF combination in accurately reflecting the influent characteristics in the treated water.

Strong positive correlations were also observed between distinct parameters, namely COD inf vs. BOD eff (r = 0.78) and COD eff vs. BOD inf (r = 0.74), suggesting that an increase in organic pollutants measured by COD corresponds to higher levels of BOD_5_.

Moderate positive correlations were found between TSS eff vs. TS inf (r = 0.66) and TSS eff vs. TS eff (r = 0.60), indicating that the presence of suspended solids is associated with higher concentrations of total solids in the water samples. Additionally, weaker positive correlations were identified between COD inf and TSS inf (r = 0.48), COD eff and TSS inf (r = 0.34), TS eff and COD inf (r = 0.43), and TS eff and COD eff (r = 0.37). These findings suggest that elevated levels of organic pollutants coincide with increased amounts of both suspended and total solids in the water.

In terms of microbiological parameters, a weak positive correlation was observed between TS eff and HE inf (r = 0.25), indicating a potential association between organic pollution and the presence of helminthes eggs. However, no significant correlation was found between TCOL and HE, despite similar removal efficiencies of the SDB and VSSF. This lack of correlation may be attributed to fluctuations in the counts of both parameters from influent to effluent.

Overall, these correlation findings highlight the interconnected nature of water quality parameters and provide insights into their relationships within the treatment system.

## Conclusions

By optimizing the design and operational parameters of each system and considering the specific requirements of the treatment site, the combined approach of Sludge Drying Bed-VSSF (Vertical Flow Constructed Wetland) holds great potential for achieving high levels of pollutant and pathogen removal. The Vertical Flow Constructed Wetland system, equipped with pretreatment by sludge drying bed, has proven to be efficient in achieving impressive removal rates. In septage wastewater, it demonstrated removal rates of 88% for BOD_5_, 87% for COD, and 65% for TSS. Additionally, the CW (Constructed Wetland) system exhibited significant effectiveness in reducing microbial contamination, consistently achieving an average removal efficiency of 99% for both total coliforms and helminthes eggs. These results not only showcase the efficiency of the combined SDB-VSSF approach but also highlight its contribution to sustainable and environmentally friendly practices.

## Data Availability

All experimental results have been included in the manuscript. The data can be made available upon reasonable request to the corresponding author.
